# Different Approaches for the Preparation of Composite Ionic Liquid-Based Membranes for Proton Exchange Membrane Fuel Cell Applications—Recent Advancements

**DOI:** 10.3390/membranes13060593

**Published:** 2023-06-11

**Authors:** Mohammad Ebrahimi, Kateryna Fatyeyeva, Wojciech Kujawski

**Affiliations:** 1 Polymères Biopolymères Surfaces (PBS), INSA Rouen Normandie, University Rouen Normandie, UMR 6270 CNRS, 76000 Rouen, France; 2 Faculty of Chemistry, Nicolaus Copernicus University in Toruń, 87-100 Toruń, Poland

**Keywords:** proton exchange membrane fuel cell, ionic liquid, polymer electrolyte membrane, incorporation, impregnation, cross-linking

## Abstract

The use of ionic liquid-based membranes as polymer electrolyte membranes for fuel cell applications increases significantly due to the major features of ionic liquids (i.e., high thermal stability and ion conductivity, non-volatility, and non-flammability). In general, there are three major methods to introduce ionic liquids into the polymer membrane, such as incorporating ionic liquid into a polymer solution, impregnating the polymer with ionic liquid, and cross-linking. The incorporation of ionic liquids into a polymer solution is the most common method, owing to easy operation of process and quick membrane formation. However, the prepared composite membranes suffer from a reduction in mechanical stability and ionic liquid leakage. While mechanical stability may be enhanced by the membrane’s impregnation with ionic liquid, ionic liquid leaching is still the main drawback of this method. The presence of covalent bonds between ionic liquids and polymer chains during the cross-linking reaction can decrease the ionic liquid release. Cross-linked membranes reveal more stable proton conductivity, although a decrease in ionic mobility can be noticed. In the present work, the main approaches for ionic liquid introduction into the polymer film are presented in detail, and the recently obtained results (2019–2023) are discussed in correlation with the composite membrane structure. In addition, some promising new methods (i.e., layer-by-layer self-assembly, vacuum-assisted flocculation, spin coating, and freeze drying) are described.

## 1. Introduction

**Figure 1:**
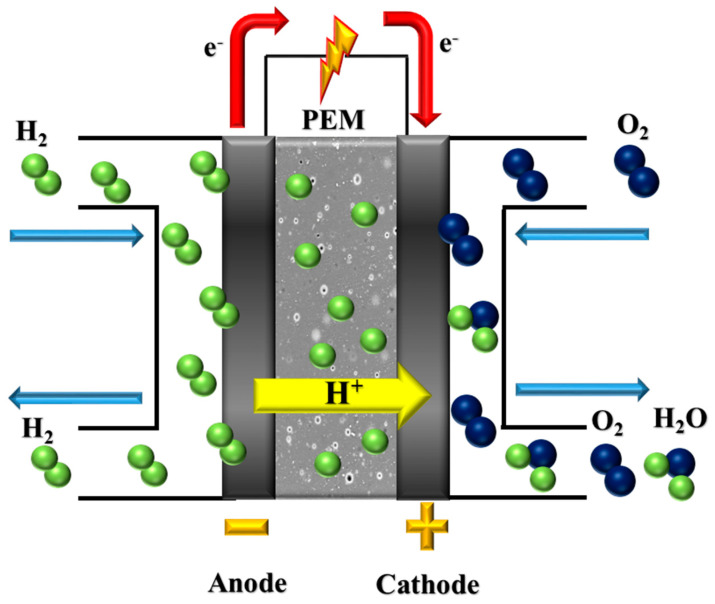
The typical schematic of a proton exchange membrane fuel cell (redrawn from [27]).

The generation of energy is one of the most crucial challenges for humans, especially for future generations [1,2]. In general, there are two main resources for generating energy: non-renewable resources and renewable ones [3,4]. The non-renewable or classical resources of energy production usually include burning fossil fuels or using nuclear energy which cause uncountable serious environmental dangers (e.g., radioactive waste) and health problems [5,6,7]. The most dangerous one is global warming, which has a domino effect on our lives [8,9]. Therefore, an interest in using green alternatives for energy generation has increased in recent decades [10,11]. A wide range of ecofriendly resources are being employed to generate green energy, including geothermal, solar, wind, hydropower, and fuel cell, of which fuel cell technology has been used significantly both in laboratories and at industrial scales [12,13,14]. Indeed, the widespread use of fuel cells is owing to its unique properties, including high efficiency, quick start-up, and desirable power density [12,13,14,15,16]. A fuel cell is an electrochemical device capable of generating electricity from chemical energy [17]. Up to now, various types of fuel cells have been invented and used according to the electrolyte type, operating condition, and type of fuel, namely, direct methanol fuel cell (DMFC), molten carbonate fuel cell (MCFC), solid oxide fuel cell (SOFC), alkaline fuel cell (AFC), phosphoric acid fuel cell (PAFC), and proton exchange membrane fuel cell (PEMFC) [18,19]. Specifically, PEMFC has gained a lot of attention among scientists, which is observable by the rising number of published articles [20,21]. Furthermore, PEMFC is being utilized in different applications such as portable devices, transportation, and residential back-up power generation [1,22,23,24]. Generally, each PEMFC consists of several parts including the polymer electrolyte membrane (PEM), current collector, catalyst, end plate, bolts, gas diffusion layer (GDL), gasket, and field flow channel (FFC) [20,24]. The most vital part that can influence the PEMFC’s performance and efficiency is the PEM, owing to the fact that the membrane acts as the transporter agent of hydrogen ions (protons) from the anodic section to the cathodic section (Figure 1) [20,24,25,26].

**Figure 2:**
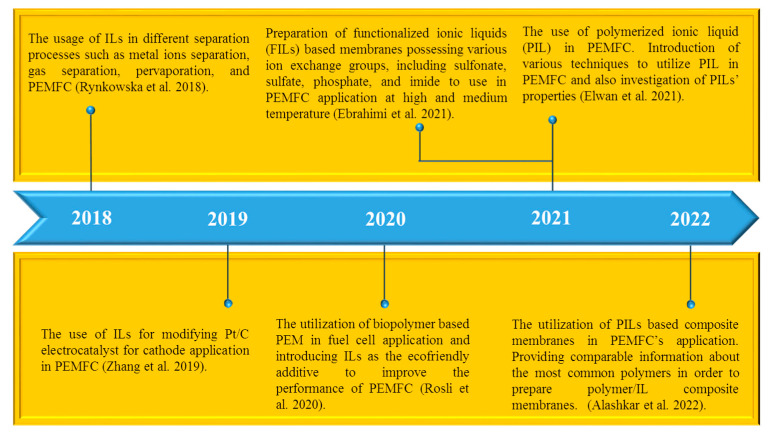
Timeline showing the published review articles concerning ILs using in separation processes, specifically their use in PEMFC (based on [27,32,34,47,48,49]).

Typically, perfluoro-sulfonated polymers (PSP) are the most common polymers (i.e., Nafion^®^, Flemion^®^, and Hyflon^®^) for the application of the PEM since they are ionic conductive and are mechanically, chemically, and electrochemically stable [28,29,30,31]. Nonetheless, PSPs’ performance is strongly dependent on humidity, as water acts as the proton carrier (mobile phase) [32,33]. However, at temperatures higher than 90 °C, water evaporates and proton conduction reduces significantly [32,33]. Many attempts have been made thus far to find desirable alternatives for obtaining the PEM which is able to be utilized in the low to high temperature range [34,35,36]. Composite membranes containing nanoparticles, inorganic compounds, and ionic liquids (ILs) demonstrate promising performance for being utilized in PEMs [37,38,39]. However, the use of IL-based composite membranes as PEMs has significantly increased compared to the past (especially in the last decade) owing to their considerable thermochemical features [34,39,40]. Indeed, ILs are chemical compounds possessing cations (organic) and anions (organic or inorganic) with specific features such as high ionic conductivity, low volatility, low toxicity, good thermal stability, and non-flammability [41,42,43,44]. Therefore, IL-based membranes can be a promising alternative for use as a PEM component at low, middle, and high temperature [32,39]. Stack preparation using IL-based membranes for PEMFC industrial application attracts much attention due to the high power density and low generated contamination [45,46]. However, there are some serious obstacles for scaling up PEMFC which are mainly related to the high cost of the catalyzer (e.g., platinum, palladium, and iridium), ILs, and pure hydrogen. The price of a PEMFC stack might be optimized by modifying the design and nature of the materials [45,46]. Up to now, a number of review articles related to IL application in separation processes, especially their use in PEMFC, have been published [32,34,39]. Rynkowska et al. [47] provided a general review about the usage of ILs in various separation processes including metal ion separation, gas separation, pervaporation, and PEMFC. Zhang et al. [48] published a review paper with regard to the use of ILs for modifying Pt/C electrocatalysts for cathode application in PEMFC. In that review article, the impact of IL content, dispersing solvent, and adsorption time was discussed [48]. Rosli et al. [49] presented a review study related to a biopolymer-based PEM in fuel cell applications and introducing ILs as the ecofriendly additive for improving the performance of PEMFC. Ebrahimi et al. [27] presented a review on the preparation of functionalized ionic liquids (FILs)-based membranes possessing various ion exchange groups, such as imide, phosphate, sulfonate, and sulfate, to use in PEMFC applications at high and medium temperatures. Different techniques of IL synthesis, the leaching phenomenon from IL-based composite membranes, and the methods to diminish it were also discussed [27]. Elwan et al. [34] published a review article concerning the use of polymerized ionic liquids (PILs) in PEMFC. In that work, various techniques to utilize PILs in PEMFC and also the investigation of PILs’ properties (i.e., conductivity and thermomechanical features) were discussed [34]. Alashkar et al. [32] prepared a review study focused on the utilization of PIL-based composite membranes in PEMFC’s application and introduced the common methods of the preparation of PILs. Furthermore, they provided comparable information about the most common polymers in order to prepare polymer/IL composite membranes [32]. Figure 2 demonstrates a timeline related to published review articles regarding the use of ILs in different separation processes, particularly in PEMFC.

Several review studies about IL use in PEMFC processes have been already published [27,32,34,47,48,49]. However, to our knowledge, no review about the method of the IL introduction into the polymer membrane exists. It is known that the IL structure and its distribution within the polymer matrix will significantly influence the composite membrane properties. In the present review, the most commonly used techniques for the elaboration of IL-based polymer membranes for fuel cell applications (i.e., IL introduction into the polymer solution, the polymer impregnation by IL, and cross-linking) are given and discussed in detail. In addition, new methods (such as layer-by-layer self-assembly, vacuum-assisted flocculation, spin coating, and freeze drying) were described.

## 2. Incorporation of IL into a Polymer Solution

**Figure 3:**
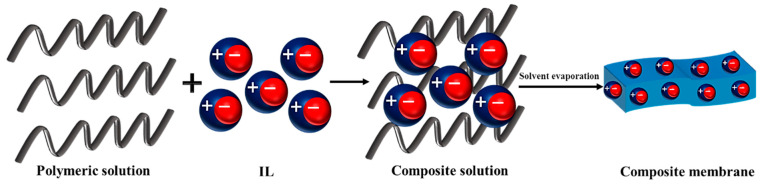
The schematic of membrane preparation by incorporation of IL into a polymer solution.

The incorporation of IL into a polymer solution is the most common technique of IL introducing into polymer film during the preparation of IL-based composite membranes. In this method, IL is dissolved or dispersed into a polymer solution that includes polymer and solvent (Figure 3). The physicochemical features of the ion gel solution strongly depend on the IL and polymer ratio. Moreover, the compatibility between IL and polymer can influence the properties of the composite membrane. Even though the ion gels have great thermal stability and excellent proton conductivity, a reduction in the mechanical stability is the main drawback of this method since the presence of IL in the composite film leads to a change in the molecular structure (spatial orientation) and in the bonding of the polymer. Therefore, there is a limitation in using of this method for the addition of IL into a polymer solution. In other words, by increasing the content of IL, ionic conductivity increases while the film’s mechanical stability diminishes. Another limitation of this method is the IL leakage at high concentrations. In fact, owing to the macromolecular movements of polymer chains, the extra amount of IL is released from the membrane.

Trindade et al. [50] prepared sulfonated poly(ether ether ketone) (SPEEK)-based composite membranes by incorporation of three imidazolium-based ILs ([Im][HSO_4_], [MI][HSO_4_], and [BMI][HSO_4_]) containing three different cations (imidazolium, 1-methylimidazolium, and 1-butyl-3-methylimidazolium) and hydrogen sulfate as the anion. The preparation protocol was to stir and dissolve both SPEEK and IL into the *N,N*-methylpyrrolidone (NMP) as the solvent at 80 °C. Subsequently, the polymer solution was cast on a glass plate and finally dried at 80 °C under vacuum for 3 days. In that study, composite membranes with 5, 10, and 15 wt.% of ILs were prepared. The results showed that the rise in temperature from 25 to 80 °C led to the enhancement of the proton conductivity for both pure SPEEK and ILs/SPEEK, and the highest value (150 mS·cm^−1^) was observed for the sample containing 5 wt.% of [MI][HSO_4_] at 80 °C and 60% of relative humidity (RH). Results revealed that there is a desirable compatibility between ILs and polymer and the addition of ILs to the membrane caused the decrease in the membrane surface roughness. However, the resultant membranes did not demonstrate great thermal stability revealing that these membranes cannot be good candidates for use as PEMs for high-temperature PEMFC applications. In other research, Elumalai et al. [30] prepared composite membranes containing phosphonated IL-Santa Barbara Amorphous-15 (SBA-15)/SPEEK to use as PEMs for PEMFC application at an elevated temperature. For composite membrane preparation, SPEEK was dissolved in the solvent until the homogenous polymer solution was obtained, and then various concentrations of phosphonated IL-SBA-15 (2, 4, 6, and 8 wt.%) were added to the mentioned solution. Stirring and sonication of the solution for 48 h and 30 min, respectively, were the next steps of preparation protocol. The composite solution was cast on a Petri dish and heated in an oven for 8 h to evaporate the solvent. The results showed that the addition of phosphonated IL-SBA-15 led to increasing water uptake owing to the fact that the presence of additives caused the formation of gaps and cavities in the membrane. Moreover, the composite samples exhibited a higher tensile strength in comparison with the pure SPEEK membrane, and the maximum value of 23 MPa was observed for a composite sample containing 6 wt.% of IL-SBA-15. However, by further increasing IL from 6 to 8 wt.%, the mechanical stability decreased. In addition, the conductivity of the samples (both pure and composite samples) was enhanced by increasing the temperature from 60 to 140 °C, and the composite membrane containing 6 wt.% of phosphonated IL-SBA15 exhibited the excellent conductivity of 10.2 S·cm^−1^ at 140 °C. Trindade et al. [51] prepared a new composite PEM based on SPEEK modified by a metal organic framework (MOF) and ILs. In that research, they encapsulated three ILs ([TEA-PS][HSO_4_], [BImH][HSO_4_], and [BMI][HSO_4_]) into the framework of UiO-66. The ILs contained three various cations (N,N,N-trietyl-3-sulfinopropan-1-aminium, 1-butylimidazolium, and 1-butyl-3-methylimidazolium) and hydrogen sulfate as the common anion. For this purpose, 0.5 g of sulfonated polymer was dissolved into the *N,N*-dimethylacetamide (DMA); subsequently, ILs (2.5 or 5 wt.%)/UiO-66 (7.5 wt.%) were added to the polymer solution and stirred for 8 h at 80 °C. In the next steps, casting and drying (at 80 °C for 24 h) of the composite solutions were carried out. The results regarding the ion exchange capacity (IEC) showed that this parameter was reduced by increasing the amount of ILs from 2.5 to 5 wt.% owing to the probable interactions between the ILs’ cations and the –SO_3_H group in the polymer body. The highest and lowest IEC values were reported for [TEA-PS][HSO_4_]—2.5 wt.%/UiO-66/SPEEK and [BImH][HSO_4_]—5 wt.%/UiO-66/SPEEK—1.78 and 1.52 mmol·g^−1^, respectively. Additionally, the proton conductivity of the samples was evaluated at two operating conditions (25 °C/100% RH and 80 °C/60% RH). The results showed that the increase in temperature from 25 to 80 °C resulted in a rise of the conductivity of all membrane samples, and the maximum value was reported for [TEA-PS][HSO_4_]—2.5 wt.%/UiO-66/SPEEK (140 mS·cm^−1^ at 80 °C and 60% RH). Yilmazoglu et al. [52] prepared composite membranes containing sulfonated polysulfone (SPSU) and ILs. Three different triazole-based ILs (TIL_1_, TIL_2_, and TIL_3_) were synthesized and utilized in this research with two cations (3,5-dibutyl-1-methyl-3H-1,2,3-triazolium and 1-benzyl-3,5-dibutyl-3H-1,2,3-triazolium) and three anions (trifluoromethanesulfonate, tetrafluoroborate, and bromide). In order to prepare the composite membrane, the SPSU was dissolved in NMP and continuously stirred for 2 h. Subsequently, various concentrations of the synthesized ILs were added to the polymer solution and stirred for 4 h. The resultant composite solution was cast and heated for 24 h at 40 °C to remove the solvent. Moreover, thermogravimetric analysis (TGA) demonstrated that the TILs/SPSU composite membrane samples were thermally stable at an elevated temperature. It was found that raising the temperature from 105 to 175 °C brought about the presence of an upward trend for proton conductivity of the composite membranes, and the TIL_3_/SPSU composite membrane illustrated the best proton conductivity of 5.81·10^−2^ S·cm^−1^ at 175 °C, while the pure SPSU membrane at the same operating condition demonstrated the lowest value (8.05·10^−3^ S·cm^−1^). The obtained results showed that IL incorporating into the polymer solution led to enhancing of the proton conduction through the SPSU membrane. In another study, Rogalsky et al. [53] fabricated composite membranes by incorporating various concentrations (30, 40, 50, and 60 wt.%) of hydrophobic protic ionic liquid (PrIL) ([BAIM][TFSI]) with the cation of 2-butylaminoimidazolinium and the anion of bis(trifluoromethylsulfonyl)imide into the polyimide (PI) Matrimid^®^ polymeric solution. For this purpose, 5 g of polymer was dissolved in 100 mL of methylene chloride (solvent); then, different contents of [BAIM][TFSI] were introduced into the resultant polymer solution. In the next step, the composite solution was cast on a glass plate and dried at RT overnight; then, to remove the traces of the methylene chloride from the membrane, it was heated at 60 °C for 12 h under vacuum conditions. The [BAIM][TFSI] showed good proton conductivity (5.6·10^−2^ S·cm^−1^ at 140 °C) and thermal stability (up to 350 °C). Furthermore, the [BAIM][TFSI]/PI composite membranes were thermally stable in the temperature range of 377–397 °C. Moreover, the tensile strength of the modified membrane possessing 30 wt.% of PrIL was higher than that of the pure PI membrane (72.5 and 37 MPa, respectively), while by increasing the PrIL from 30 to 60 wt.%, the tensile strength demonstrated a downward trend in which the lowest value was observed for composite membrane containing 60 wt.% of [BAIM][TFSI] (16 MPa). The proton conductivity results showed that raising the operation temperature from 25 to 140 °C led to a considerable increase in the proton conductivity of the membrane samples. For instance, regarding the composite membrane with 50 wt.% of PrIL, the proton conductivity increased from 3.1·10^−6^ to 6.3·10^−3^ S·cm^−1^ by enhancing the temperature from 25 to 140 °C. Additionally, the increase in the concentration of PrIL led to a rise in proton conductivity. Trindade et al. [54] fabricated a new composite membrane containing IL/polybenzimidazole (PBI)/SPEEK. Two ILs ([TEA-PS][HSO_4_] and [BImH][HSO_4_]) were employed with the same anion (hydrogen sulfate) and different cations (N,N,N-trietyl-3-sulfinopropan-1-aminium and 1-butylimidazolium) to evaluate the impacts of different cations on the features of composite membranes. In that research, to prepare the composite solution, first of all, the SPEEK was added to 1 M NaOH solution for 24 h until the pH of 7 was reached. The formed SPEEKNa, PBI, DMA, and [TEA-PS][HSO_4_] or [BImH][HSO_4_] (2.5 and 5 wt.%) were mixed and stirred together at 80 °C. The resultant composite solution was cast on a Petri dish and dried for 24 h at 80 °C. The membrane proton conductivity was investigated at two different operating conditions (at 25 °C and 100% RH, and at 80 °C and 60% RH). Among all composite samples, the membrane containing 10 wt.% of polymer and 5 wt.% of [TEA-PS][HSO_4_] demonstrated the maximum conductivity of 101 mS·cm^−1^ at 80 °C and 60% RH. Moreover, owing to the hydrogen bonds between ILs and PBI, the leaching of ILs reduced significantly. The highest values regarding the open circuit potential (OCP) and current density (0.97 V and 1.83 A·cm^−2^, respectively) were observed for the composite membrane with 5 wt.% of [TEA-PS][HSO_4_] and 10 wt.% of PBI. Skorikova et al. [55] fabricated composite membranes by incorporating two PrILs (diethylmethylammonium bis(trifluoromethanesulfonyl)amide, [dema][NTf_2_] and tetramethylguanidine bis(trifluoromethanesulfonyl)amide, [HHTMG][NTf_2_]) into PBI. For this purpose, 7 wt.% of PBI was dissolved in dimethylacetamide (DMAC) at the temperature of 160 °C for 24 h. In the next step, the PrIL ([dema][NTf_2_] or [HHTMG][NTf_2_]) was added to the polymer solution (50 and 67 wt.%), and the composite solution was stirred for another 24 h. Casting and drying membranes at 80 °C overnight were the next steps in the membrane elaboration. Furthermore, some of the samples were doped (i.e., immersed in an acid solution) in phosphoric acid (PA) to increase the conductive channels in the membranes. The membrane conductivity was measured in the temperature range from 80 to 180 °C. The PA-doped and undoped [HHTMG][NTf_2_]/PBI composite membranes with 67 wt.% of IL showed the highest proton conductivity (~60 and 0.3 mS·cm^−1^ at 180 °C, respectively). Moreover, based on the TGA results the composite membranes were thermally stable up to 250 °C. However, it was found that the tensile strength and Young’s modulus of composite membranes decreased in comparison with the pure PBI membrane. By increasing the amount of PrILs (from 50 to 67 wt.%), the mechanical properties of the composite membranes reduced even further. In another investigation, Rosli et al. [56] prepared composite membranes containing *N*-methylene phosphonic chitosan (NMPC)/poly(vinyl alcohol) (PVA) containing propylammonium nitrate (PAN) IL and/or silicon dioxide (SiO_2_) filler via solution casting method. The SiO_2_ powder was firstly dissolved in the PAN IL and then added to the NMPC/PVA composite solution. Samples with various amounts of PAN IL (5, 10, 15, and 20 wt.%) and 4 wt.% of SiO_2_ were prepared; subsequently, the composite solutions were cast on a Petri dish and heated during 4 days at 60 °C. The proton conductivity of the resultant membranes was evaluated at various operating temperatures (25, 40, 60, 80, and 100 °C), and the PAN IL/NMPC/PVA composite membrane containing 20 wt.% of PAN IL showed the highest proton conductivity of 1.54·10^−3^ S·cm^−1^ at 100 °C. Moreover, the highest values concerning water uptake, swelling area, and IEC were reported for PAN IL/NMPC/PVA containing 20 wt.% of PAN IL (60.5 ± 4.5%, 43.9 ± 7.1%, and 0.60 ± 0.01 milliequivalent (meq)·g^−1^, respectively). Furthermore, the TGA results showed that for composite samples there was any considerable thermal degradation until 100 °C. Additionally, the mechanical stability of membranes containing PAN IL/NMPC/PVA was lower than that of SiO_2_/NMPC/PVA membranes. 

Niu et al. [35] prepared composite PBI-based membranes by introducing various ILs for improving the electrochemical properties of PEMs for PEMFC applications at elevated temperatures. Different ILs ([C_1_Im][NTf_2_]), [dema][TfO], [emim][TfO], and [HOemim][NTf_2_]) using four cations (1-methylimidazolium, diethylmethylammonium, 1-ethyl-3-methylimidazolium, and 1-(2-hydroxyethyl)-3-methylimidazolium) and two anions (bis (trifluoromethane sulfonyl)imide and trifluoromethanesulfonate) were prepared. The PBI polymer was dissolved in *N,N*-dimethylformamide (DMF) for 5 h at the temperature of 60 °C. After preparing the homogenous solution, the IL was added to the polymer solution in different weight ratio (PBI:IL: 1:0.6, 1:0.9, 1:1.2, and 1:1.5). Although the unmodified PBI membrane demonstrated the best thermal stability up to 435 °C, the composite IL/PBI membranes showed acceptable thermal stability (between 310 and 383 °C). Moreover, the membrane proton conductivity was measured in the range from 100 to 250 °C. The results regarding conductivity revealed that increasing of both the operating temperature and IL content resulted in a rise in proton conductivity. Additionally, the [dema][TfO]/PBI membrane showed the maximum proton conductivity (108.9 mS·cm^−1^ at 250 °C). Escorihuela et al. [57] prepared various PBI-based composite membranes with several ILs ([BMIM][NCS], [BMIM][Cl], [BMIM][NTf_2_], [BMIM][Br], [BMIM][BF_4_], [BMIM][I], and [BMIM][PF_6_]) containing the same cation (1-butyl-3-methylimidazolium) and different anions (thiocyanate, chloride, bis(trifluoromethylsulfonyl)imide, bromide, tetrafluoroborate, iodide, and hexafluorophosphate). To prepare the membrane, 10 wt.% of PBI powder was dissolved in DMAC; after complete blending, 5 wt.% of IL was added to the polymer solution and stirred for 4 h at 60 °C and finally dried. The TGA results showed that the pure PBI membrane had higher thermal stability than the IL/PBI membranes. However, the resultant modified membranes showed acceptable thermal stability up to 200 °C. Moreover, the mechanical features (Young’s modulus and tensile stress) of the composite membranes were better than those of the pure PBI membrane—the highest Young’s modulus and tensile stress were reported for [BMIM][Cl]/PBI membrane (3.7 GPa and 141 MPa, respectively). Furthermore, the resultant composite membranes were characterized by good conductivity, and the maximum value was observed for [BMIM][BF_4_]/PBI (94 mS·cm^−1^ at 200 °C). Vázquez-Fernández et al. [58] fabricated an IL/poly(vinylidene fluoride) (PVDF) composite membrane by incorporation of IL (bis(2-ethyl hexyl) ammonium hydrogen phosphate [EHNH_2_][H_2_PO_4_] and imidazolium hexanoate [Im][Hex]) into PVDF. Composite membranes containing various IL concentrations (10, 20, 30, 40, and 50 wt.%) were prepared by thermally induced phase separation method using dimethyl sulfoxide (DMSO) and dimethyl carbonate (DMC) as solvents. The proton conductivity measurements performed from 20 to 60 °C revealed that the proton conductivity was reduced for the phosphate-based composite membrane, while it increased in the case of the imidazolium-based membranes. Such a result was explained by the IL structure. The [EHNH_2_][H_2_PO_4_]/PVDF composite membrane with 50 wt.% of IL showed the highest proton conductivity of 150 mS·cm^−1^ at 20 °C. Based on the reported results, it can be concluded that phosphate-based composite membranes are better candidates for low-temperature PEMFC, while imidazolium-based membranes are more suitable for middle- and high-temperature applications. 

Nair et al. [59] prepared PVDF-*co*-hexafluoropropylene (HFP)-based membranes with different diethylmethylammonium trifluoromethanesulfonate ([dema][TfO]) content for use in the low- and middle- temperature range. In this case, 40 wt.% of PA acid was added to the PVDF-HFP solution in acetone and different amounts of [dema][TfO] (20, 40, 60, and 80 wt.%) were introduced thereafter. The membranes were elaborated by solution casting method and dried in the vacuum oven. The obtained membranes showed good thermal stability (up to ~360 °C) which was, however, lower than that of the pure membrane (~490 °C). It was found that the increase in IL concentration did not significantly increase the membrane proton conductivity—the highest proton conductivity (of ~6·10^−4^ S·cm^−1^ at RT) was measured for the composite membrane with 40 wt.% of [dema][TfO]. This result was explained by IL leakage at a concentration higher than 40 wt.%. Zanchet et al. [60] prepared Nafion^®^-based composite membranes through incorporation of ammonium-based ILs with the same cation (3-triethylammonium propane sulfonic ([TEA-PS])) and different anions (tetrafluoroborate ([BF_4_]), hydrogen sulfate ([HSO_4_]), and trifluoromethanesulfonate ([CF_3_SO_3_])). Both Nafion^®^ and IL were dissolved in DMF at 80 °C during 2 h, and the obtained solution was cast on a Petri dish and dried at 80 °C overnight. In order to remove the residual DMF, membranes were additionally dried at 125 °C for 2 h under vacuum. Composite IL/Nafion^®^ membranes containing different IL concentrations (2.5, 5, and 10 wt.%) were fabricated. The proton conductivity of the composite membranes was measured at 25 and 80 °C under humid conditions. The [TEA-PS][HSO_4_]/Nafion^®^ membrane containing 5 wt.% of IL showed the highest proton conductivity at both temperatures—1.1 S·cm^−1^ at 25 °C and 1.6 S·cm^−1^ at 80 °C. It was found that IL leaching from the composite membrane was observed with a temperature rise and an increase of the IL concentration. The resultant membranes showed good potential to be used in PEMFC at low and middle operating temperatures. Table 1 provides a summary concerning the preparation of composite membranes by the incorporation of ILs into a polymer solution.

## 3. Impregnation of the Polymer with IL

**Figure 4:**
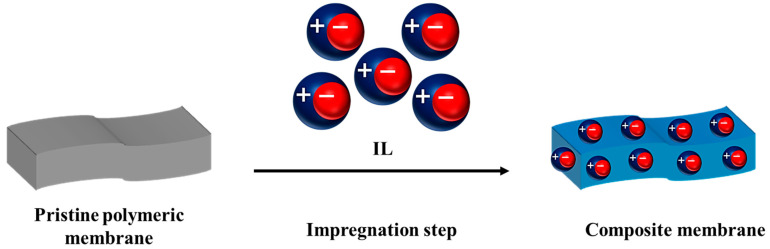
The schematic of membrane preparation by impregnating of the polymer with IL.

The basis of polymer impregnation with IL is that the prepared pristine polymer film is immersed in the IL solution. In this technique, ILs can be located in the membrane (i.e., in pores, gaps, and cavities); therefore, the resultant composite membrane possesses numerous conductive sites for proton transport (Figure 4). The pristine polymer film can be fabricated using a simple phase inversion technique, and it can possess either dense or porous (micro- or nanoporous) structure as a function of user requirements. Both homopolymers (with a single kind of monomer) and copolymers (possessing two different monomers) can be used for this approach. The interactions between polymer and IL are considered not to be significant since the IL is trapped in the polymer structure by physical forces. Furthermore, absorbing IL into the polymer film is dependent on the available gaps, cavities, and free volume in the pristine polymer film and the physical affinity between polymer and IL; consequently, the quantity of adsorbed IL is limited. The polymers impregnated by IL typically showed good proton conductivity. However, the use of this method is not strongly recommended because the leaching of unbound IL is considerable, and this method is not affordable from an economic point of view.

Fatyeyeva et al. [61] prepared a composite PrIL/Matrimid^®^ membrane for PEMFC application. The porous Matrimid^®^ film was manufactured using the vapor-induced phase separation (VIPS) technique. The polymer solution contained 14 wt.% of Matrimid^®^, 7 wt.% of polyvinylpyrrolidone (PVP) as porogen agent, and NMP as solvent. The resultant porous membranes were swelled in the 10 g of PrIL at 60 °C for 24 h for good penetration of PrIL into the membrane pores. The solution was slowly cooled to room temperature. In that study, four various PrILs with the same anion (bis(trifluoromethylsulfonyl)imide) and different cations (1-methylimidazolium, 1-ethylimidazolium, 1-propylimidazolium, and 1-butylimidazolium) were utilized (PrILs: [MIM][TFSI], [EIM][TFSI], [PIM][TFSI], and [BIM][TFSI], respectively). It was found that there was a direct correlation between proton conductivity and temperature – the composite films showed good proton conductivity of around 1.0·10^−3^ S·cm^−1^ at a high temperature. Additionally, the resultant composite PrIL-based membranes showed excellent thermal stability (between 260 and 290 °C). Furthermore, by introducing the PrIL into the membrane, the mechanical stability enhanced in comparison with pristine polymer membrane owing to the probable (physical) interactions between the imidazolium ring of the PrIL and polymer chains. In another work, Dahi et al. [62] fabricated a new supported ionic liquid membrane (SILM) in which the porous PI membrane was impregnated by three different PrILs ([C_4_im][BEHP], [C_4_im][DBP], and [C_1_im][DBP]) containing two cations (1-*n*-butylimidazolium and 1-*n*-methylimidazolium) and two anions (bis(2-ethylhexyl)phosphate and dibutylphosphate). The porous PI membrane was elaborated via VIPS technique. After drying the membrane, it was submerged in the PrIL solution at 25 °C for a specified time until the PrIL penetration was completed. The resultant membranes contained 53 ± 3% of PrIL. The PrIL-based membranes demonstrated a great retention ability against losing the ILs owing to the desirable interactions between the polymer and PrILs. Membrane proton conductivity was also evaluated in the temperature range between 25 and 115 °C. The highest conductivity was reported for the composite membrane with [C_4_im][DBP] (2.0·10^−2^ S·cm^−1^ at 115 °C). Kobzar et al. [63] prepared a new SILM possessing polyoxadiazole and methyl imidazolium trifluoromethane sulfonate ([MeIm][Tf]) using a solution casting technique. A dense polyoxadiazole/PVP (50/50, *v*/*v*) composite membrane was first prepared. Then PVP was removed by washing in the mixed water/methanol solution in order to form in the membrane extra gaps and cavities. After the complete drying of the resultant membrane, it was immersed in the aqueous solution containing [MeIm][Tf] and dried again. The impregnation of the polymer with IL was repeated several times for each membrane. The proton conductivity of the composite sample was measured in the temperature range between 40 and 120 °C; by increasing the operating temperature, proton conductivity showed an upward trend. The resultant composite membranes showed a remarkable degree of IL impregnation of 297%, thus ensuring a high ion conductivity of 1.3·10^−3^ S·cm^−1^ at elevated temperatures and under non-humid conditions. It was found that there was no thermal degradation up to 350 °C. Moreover, the impregnated membranes demonstrated good mechanical features such as Young’s modulus and elongation at break of 0.3 to 21.4 MPa and 6 to 174%, respectively. In another research study, Kobzar et al. [64] prepared composite membranes based on ILs and polyimide Matrimid^®^ polymer. The elaboration of composite membranes was performed by impregnation of porous polyimide membranes with three different ILs and one polymerized IL (PIL) – vinyl imidazolium trifluoromethane sulfonate [VIm][Tf], allyl imidazolium trifluoromethane sulfonate [AIm][Tf], methacrylate imidazolium trifluoromethane sulfonate [MIm][Tf], and polymerized vinyl imidazolium trifluoromethane sulfonate [PVIm][Tf]. The porous polyimide membrane was immersed for 12 h in IL. In case of PIL, the membranes were immersed in an aqueous solution containing water and PIL (66.7 wt.% PIL). The impregnation content of [VIm][Tf]/PI, [AIm][Tf]/PI, [PVIm][Tf]/PI, and [VIm][Tf]/[PVIm][Tf]/PI membrane samples was 210, 170, 146, and 276 wt.%, respectively. The resultant composite membranes exhibited good mechanical features such as Young’s modulus and elongation at break (1371 MPa and 271%, respectively). Moreover, thermal analysis revealed that there was no significant thermal degradation up to 300 °C. The composite films showed acceptable conductivity at both low and elevated temperatures (between 10^−2^ and 10^−1^ mS·cm^−1^). 

Al-Othman et al. [65] conducted research on composite polymer films containing zirconium phosphate (ZrP), 1-ethyl-3-methylimidazolium ethyl sulfate ([EMIM][ESO_4_]), and polytetrafluoroethylene (PTFE). In this study, an alcohol suspension was prepared firstly by dissolving zirconium oxychloride in water and isopropanol, then [EMIM][ESO_4_] and glycerol were added to the suspension and stirred for specified time. Subsequently, the porous PTFE support was filled by the mentioned suspension, and then PA was added to produce ZrP. Washing with water and drying for 8 h at 90 °C were the next steps. The resultant [EMIM][ESO_4_]/ZrP/PTFE membrane presented a great proton conductivity of 61 mS·cm^−1^ at 200 °C and under dry conditions. The composite film was thermally stable, showing only 20% weight loss at the temperature of 500 °C. Zakeri et al. [66] prepared a new supported dual acidic IL membrane based on poly(4-vinyl pyridine) grafted by ethylene-*co*-tetrafluoroethylene (ETFE) for PEMFC application. The grafting process was performed using an irradiation (electron beam) technique. The grafted dense ETFE film was impregnated by dual acidic IL to improve the transport features of the resultant composite film. Two membranes with two different IEC values (3.2 and 3.4 meq·g^−1^) were fabricated. The composite membrane with the IEC of 3.4 meq·g^−1^ demonstrated better proton conductivity in the temperature range from 30 to 95 °C in comparison with the membrane with the IEC of 3.2 meq·g^−1^. The composite membrane sample with the IEC of 3.4 meq·g^−1^ showed a proton conductivity of 259 mS·cm^−1^ at 95 °C and under fully hydrated conditions, that was higher than the value of Nafion^®^112 (160 mS·cm^−1^). The resultant composite membrane is characterized by great thermal stability up to 280 °C and by better mechanical and chemical stability in comparison with Nafion^®^112. Javed et al. [67] prepared a composite membrane containing zirconium silicate (ZrSi), glycerol (GLY), IL (1-hexyl-3-methylimidazolium tricyanomethanide [Hmim][TCM], 1-butyl-3-methylimidazolium thiocyanate [Bmim][SCN]), and porous PTFE for application in PEMFC at low and high temperatures (at 25 and 200 °C). Composite membranes with various concentrations of ILs (0.56, 0.7, 1.1, and 2.5 wt.%) and GLY (0.17, 0.2, 0.4, 0.5, 0.6, 0.75, 0.8, 1.25, 2 wt.%) were fabricated. Scanning electron microscope (SEM) images confirmed the presence of IL in the membrane’s pores. It was found that the membrane proton conductivity depended directly on the membrane water uptake. All prepared composite membranes showed high water uptake (higher than 50%) and excellent proton conductivity (of ~0.1–0.2 S·cm^−1^ at 25 °C). This conductivity value was found to be higher than that of the Nafion^®^ membrane (0.01 S·cm^−1^) at the same operating temperature. TGA analysis demonstrated a high thermal stability (up to 200 °C) of composite membranes, and only 18% of membrane were decomposed at 500 °C. Tawalbeh et al. [68] developed composite polymer films containing lignin, ZrP, IL (1-ethyl-3-methylimidazolium methanesulfonate [Emim][CH_3_O_3_S], 1-hexyl-3-methylimidazolium tricyanomethanide [Hmim][C_4_N_3_] or diethylmethylammonium methanesulfonate [DMEA][OMS]), and porous PTFE. Composite membranes with various concentrations of ILs (0.3, 0.6, 0.9, and 1.5 wt.%) were prepared through the PTFE impregnation. The proton conductivity of the composite membranes measured from 25 to 150 °C was found to increase. The [Hmim][C_4_N_3_](0.3 wt.%)/lignin/ZrP/PTFE composite membrane showed the highest proton conductivity—10^−1^–10^−3^ S·cm^−1^ at 25–150 °C. The high proton conductivity measured at 25 °C makes this membrane a promising candidate for low-temperature PEMFC application. Table 2 shows a summary concerning the preparation of composite membranes by the polymer impregnation with ILs.

## 4. Cross-Linking

**Figure 5:**
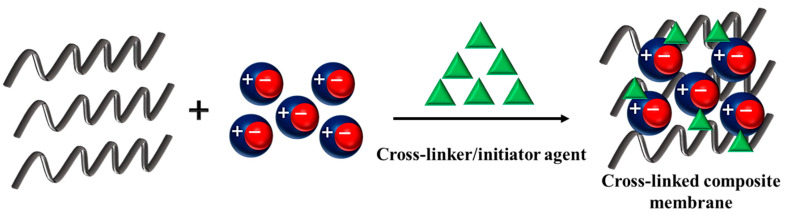
The schematic of membrane preparation by cross-linking.

Generally, the interactions between the IL and the polymer are physical interactions which are not strong enough in the two earlier mentioned methods, and the IL leaching from the polymer film is a serious issue that reduces the membrane proton conductivity during the process. IL cross-linking onto the polymer matrix can be a good technique to reduce the IL leaching. Indeed, a formation of covalent bonds between membrane components (here, IL and polymer chains) (by introducing a cross-linker) takes place during the cross-linking [69]. The main advantage of this technique is ensuring stable ionic conductivity during a long period of time due to the reduced IL leakage. However, in this case, the membrane ionic conductivity decreases owing to the reduction in the IL’s ionic mobility and the polymer chain mobility. The graphical representation of this method is shown in Figure 5.

Liu et al. [70] prepared a functional PIL cross-linked norbornene (Nb)-type PBI membrane via a solution casting method. In that research, in order to prepare the composite NbPBI/PIL membrane, polymer, PILs (P[VBIm][Cl], P[MPIm][Br], P[TPAm][Br]), and azobis-(isobutyronitrile) (AIBN) were dissolved in DMAC. The resultant composite solution was cast at 60 °C for a day and then dried at 110 °C to remove the DMAC from the membrane. The Fourier-transform infrared spectroscopy (FTIR) spectra confirmed that PILs were chemically cross-linked to the polymer and composite samples showed good resistance in DMAC. The resultant composite membranes showed an excellent proton conductivity of 74 mS·cm^−1^ at 170 °C. In addition, the composite NbPBI/PIL membranes were thermally stable in the temperature range from 220 to 250 °C. The composite membrane containing PIL demonstrated a power density of 385 mW·cm^−2^ at 160 °C. In another work, Liu et al. [71] prepared a novel type of composite film by the cross-linking reaction between NbPBI and PIL-containing trimethoxysilane groups ([TSPDO][BrCl]). In order to form cross-linked networks between NbPBI and PIL, the in situ free radical polymerization reaction was carried out. Furthermore, to form Si−O−Si networks, an in situ sol–gel reaction was performed. To enhance the conductivity of the resultant composite membranes, they were immersed in 1 M sulfuric acid at 80 °C for 24 h. The composite membrane demonstrated a great proton conductivity of 61 mS·cm^−1^ at 170 °C and showed a better thermal stability up to 300 °C (for comparison, it was 250 °C for pristine NbPBI film). The tensile strength and elongation at break of samples were measured before and after PA doping (i.e., immersion of membrane in PA solution). Both pristine and composite membranes showed excellent tensile strength before PA doping (between 116 and 125 MPa), and the composite membrane exhibited better elongation at break compared with the pristine membrane (18 ± 1% and 10 ± 1%, respectively). However, owing to the breaking of the hydrogen bonds after doping, the mechanical features of the membranes diminished considerably. The composite membranes revealed higher values of both tensile strength and elongation at break as compared with the pristine NbPBI membrane due to the double cross-linked bonds. Lin et al. [72] studied a new hybrid membrane containing poly(styrene/acrylonitrile) (PSAN)/divinylbenzene/1-methylimidazolium trifluoromethanesulfonate ([MIm][TfO])/1-(3-aminopropyl)-3-methylimidazolium bromide functionalized graphene oxide ([APMIm][Br]-GO). The mentioned polymer and ILs were mixed and stirred together in the presence of benzoin isobutyl ether as the photo-initiator agent in order to obtain the homogeneous solution, and a resultant solution was then cast on a glass plate. The cast hybrid solution was photo cross-linked with UV light (irradiation step) for 30 min to form new bonds and interactions in the polymer matrix and to stabilize the ILs in membranes. The hybrid film containing 1 wt.% of [APMIm][Br]-GO demonstrated the highest conductivity of 14.8 mS·cm^−1^ at 160 °C, whereas for the membrane sample without [APMIm][Br]-GO, the conductivity was 1.4 mS·cm^−1^. The hybrid films showed good mechanical stability. Besides, by increasing the concentration of [APMIm][Br]-GO from 0 to 1.2 wt.%, the values of tensile strength and tensile modulus increased, while elongation at break are reduced as [APMIm][Br]-GO has a brittle nature. Moreover, the TGA illustrated that [APMIm][Br]-GO introducing did not have a considerable impact on the membrane thermal behavior, and all membrane (with or without [APMIm][Br]-GO) showed great thermal stability (only 9% of weight loss at the temperature of 300 °C). Furthermore, the leaching test demonstrated that [APMIm][Br]-GO introducing reduced remarkably the leakage of [MIm][TfO] from the membranes owing to the interactions between [APMIm][Br]-GO and [MIm][TfO] during the cross-linking step. In another work, Chen et al. [73] prepared new cross-linked composite membranes based on PBI and PIL. PBI and PIL (10, 20, 30, and 40 wt.%) were blended in the presence of DMAC as the solvent. Subsequently, 3 wt.% of the cross-linker agent (γ-(2, 3-epoxypropoxy) propyltrimethoxysilane) was added to the mentioned composite solution and stirred for several hours until obtaining a homogeneous solution. Afterwards, the composite solution was cast and heated (80 °C for 24 h) to remove DMAC. In the next step, the resultant polymer film was submerged in sulfuric acid (1 M), and the solution was heated for 24 h at 80 °C. In the last step, the cross-linked films were placed in an oven for 24 h at the temperature of 120 °C. Findings revealed that the increase of the operating temperature provoked the membrane proton conductivity rising, and the PBI/PIL composite film with 40 wt.% of PIL revealed maximum value (117 mS·cm^−1^ at 170 °C). The PBI/PIL composite membranes showed acceptable mechanical stability; however, by increasing the content of PIL from 10 to 40 wt.%, the mechanical stability decreased, and the composite sample with 10 wt.% demonstrated the highest tensile strength of 84.4 ± 7.6 MPa. Moreover, the resultant composite membranes exhibited great thermal stability in the range from 300 to 500 °C. Ortiz-Martinez et al. [74] studied a new composite membrane with an IL ([HSO_3_-BVIm][TfO]) containing 1-(4-sulphobutyl)-3-vinylimidazolium and trifluoromethanesulfonate as the cation and anion, respectively, and hydrolyzed perfluoro-3,6-dioxa-4-methyl-7-octene sulfonyl fluoride (hPFSVE) or methyl methacrylate (MMA). In that study, [HSO_3_-BVIm][TfO] was copolymerized by hPFSVE and MMA with various amounts of hPFSVE (10, 20, 30, and 40 mol%) and MMA (5 and 10 mol%) in the presence of 5 wt.% of glycerol dimethacrylate and 2 wt.% of 2-hydroxy-2-methyl propiophenone as cross-linker and photo-initiator agents, respectively. The resultant composite solution was cast on a glass plate and then placed under UV light for 30 min. The FTIR results confirmed that the photochemical copolymerization reaction between IL and both hPFSVE and MMA was performed. The membrane proton conductivity was measured in the temperature range from 25 to 90 °C, and the temperature rise led to enhancing the proton conductivity. Moreover, the resultant membranes showed good conductivity in both dry and wet states (in the range from 1 to 10 mS·cm^−1^). In addition, the thermal analysis displayed that the composite films had great thermal stability (≥200 °C). The composite membranes with 10 mol% of MMA and hPFSVE showed the highest power density of 45.76 and 28.12 mW·cm^−2^, respectively. Liu et al. [75] fabricated a PBI/PIL composite membrane, in which PIL ([CPDOc][Br_2_]) was chemically cross-linked onto the polymer chain. The mentioned PIL was prepared with 3-(4-chlorobutyl)-1-pentyl-4H-imidazoledium as the cation and bromide as the anion. For preparation of the cross-linked membrane, both PBI and [CPDOc][Br_2_] were dissolved in DMSO and stirred at 80 °C for 24 h. In the next step, 1 wt.% of AIBN was added to the solution as the initiator agent and stirred again. Finally, the composite solution was cast and dried at 90 °C for another 24 h. To evaluate the cross-linking reaction, the pure PBI and composite samples were immersed in DMSO. It was found that the pure membrane was totally dissolved, but the composite membranes showed are rather stable in DMSO; in addition, by increasing the content of [CPDOc][Br_2_], the remaining weight increased, revealing the improvement of the cross-linking reaction with a further addition of IL. The tensile test demonstrated that by IL introducing to the PBI membrane, the tensile strength decreased; besides, mechanical stability decreased considerably by doping the samples in PA. However, the undoped PBI/[CPDOc][Br_2_] composite membrane sample with 10 wt.% of PIL showed the best tensile strength of 60 ± 2 MPa. Membrane conductivity was evaluated in the temperature range between 110 and 170 °C. It was observed that proton conductivity increased by increasing both the temperature and PIL concentration, and the highest conductivity was observed at 170 °C for composite film with 30 wt.% IL. Moreover, the resultant PBI/[CPDOc][Br_2_] composite films showed tunable thermal stability (between 200 and 250 °C). Table 3 presents a summary regarding the preparation of composite membranes using a cross-linking technique.

Although the mentioned methods of IL introducing into polymer membranes led to the preparation of IL-based composite membranes with proper physical, thermal, and chemical stability, efforts toward the research of new techniques should be continued. For example, despite the fact that the casting method is the most used to prepare IL-based membranes owing to such benefits as easy operation, a simple process, and fast membrane formation, sometimes heterogeneous IL distribution in the membrane is observed. This issue can be diminished by increasing the time of solution mixing and/or sonication. In order to prepare multilayered IL-based membranes, some other methods can be used, including layer-by-layer (LbL) self-assembly, vacuum-assisted flocculation, spin coating, and freeze drying [76,77,78,79]. Che et al. [77] fabricated bicomponent and multicomponent membranes via LbL self-assembly technique for PEMFC applications. In this case, a negatively charged glass substrate was immersed subsequently into a solution containing polyurethane (PU), SPEEK, and 1-butyl-3-methylimidazolium ([Bmim]) cation, and into a SPEEK solution. This cycle was repeated 100 times to obtain a (SPEEK/PU/SPEEK/bmim)_100_ membrane. A (SPEEK/PU)_210_ membrane was prepared as well by dipping the glass substrate into SPEEK and PU solutions (210 times). Some other PA-doped membranes were prepared by submerging the (SPEEK/PU/SPEEK/bmim)_100_ and (SPEEK/PU)_210_ membranes in PA solutions containing 30, 40, 50, 60, and 70 wt.% of PA. The proton conductivity of the composite membranes was investigated at temperature range from 80 to 160 °C under anhydrous conditions. The results showed that (SPEEK/PU/SPEEK/bmim)_100_/PA membranes have higher proton conductivity in comparison with (SPEEK/PU)_210_/PA membranes—~1·10^−1^ S·cm^−1^ and ~6·10^−2^ S·cm^−1^, respectively, at 160 °C. The (SPEEK/PU/SPEEK/bmim)_100_/60%PA and(SPEEK/PU)_210_/60%PA showed sufficient tensile stress (2.4 and 3.2 MPa, respectively), even though the undoped composite membranes revealed higher values (7.0 and 7.5 MPa, respectively). The (SPEEK/PU/SPEEK/bmim)_100_ and(SPEEK/PU)_210_ membranes showed good thermal stability up to 190 and 225 °C, respectively. However, regarding the PA-doped composite membranes, thermal stability was considerably reduced because of the evaporation of PA molecules. Song et al. [79] elaborated a multilayered membrane via a spin-coating technique for PEMFC applications at high temperature. The membrane preparation consisted in a multi-step process, for which a glass substrate was washed with water, acetone, and 2-propanol. Then, a Kevlar nanofiber solution was spun on the glass surface at 200 rpm for 10 s, followed by immersion in a water bath for 10 s. In the next step, a solution of 1-butyl-3-methylimidazolium chloride ([Bmim][Cl]) was coated on the Kevlar nanofiber and heated in the oven at 80 °C for 10 min. A layer of polystyrene-*block*-poly(ethylene-*ran*-butylene)-*block*-polystyrene (SBES) was deposited on the Kevlar layer. The spin-coating process was repeated five times to prepare (Kevlar-[Bmim][Cl]-SBES)_5_ ((KBS)_5_). Some of the membranes were folded ((KBS)_5_-fold) and stretched with a speed of 5 mm·min^−1^ ((KBS)_5_-x (x: 10, 30, 50 times). Some PA-doped membranes were prepared by submerging the membranes in PA solution (100 wt.% of PA). It was shown that stretching and folding change the composite membrane properties (mechanical and thermal stability and proton conductivity). The (KBS)_5_ membrane showed good thermal stability up to 230 °C, while this value decreased to ~150 °C for the PA-based membranes ((KBS)_5_/PA, (KBS)_5_-fold/PA, and (KBS)_5–_50/PA). The (KBS)_5_ membrane showed a tensile stress of 16.8 ± 3.8 MPa, and this value was enhanced for folded (~19 MPa) and stretched membranes (between 22 to 33 MPa). However, the addition of PA led to a considerable reduction in tensile stress. The stretched and folded composite membranes (PA-doped and undoped) showed higher proton conductivity than (KBS)_5_-membranes. The highest proton conductivity was reported for (KBS)_5_/PA-50 at 160 °C (8.5·10^−2^ S·cm^−1^). Zhao et al. [78] prepared multilayered composite membranes via the vacuum-assisted flocculation technique for PEMFC applications at low operating temperatures. The multilayered (PVA/Kevlar/carbon nanotube oxides (OCNTs))_3_ membrane was fabricated by alternate deposition (three times) of PVA, Kevlar nanofibers, and OCNTs. In addition, PA-doped membranes were prepared by membrane immersion in PA solutions (60, 75, and 85 wt.% of PA). The (PVA/Kevlar/OCNTs)_3_/85% PA composite membrane showed rather high proton conductivity at low operating temperatures ranging from −30 to 30 °C— 2.8·10^−2^ S·cm^−1^ at −30 °C and 1.5·10^−1^ S·cm^−1^ at 30 °C. Composite membranes demonstrated excellent long-term conductivity performance — for example, the proton conductivity of (PVA/Kevlar/OCNTs)_3_/85% PA membrane stayed 1.4·10^−2^ S·cm^−1^ at 30 °C after 1900 h of testing. However, the tensile stress of (PVA/Kevlar/OCNTs)_3_ membranes decreased from 40.6 to 2.3 MPa after the PA doping. Duan et al. [76] prepared Kevlar/polyacrylamide (PAM)/1-butyl-3-methylimidazolium chloride ([Bmim][Cl]) composite membranes by a freeze-drying (fd) technique for PEMFC applications at high temperatures. For this purpose, a gel containing Kevlar, PAM, and [Bmim][Cl] was placed in the freeze dryer at −40 °C for 12 h and for another 24 h at 80 °C in the oven. PA-doped membranes were also prepared by immersion of the Kevlar-based membranes in a PA solution (85 wt.% of PA) overnight. The Kevlar/PAM/[Bmim][Cl](fd)/PA membrane revealed a great long-term proton conductivity of ~2·10^−1^ S·cm^−1^ after 270 h at 140–180 °C. The Kevlar/PAM/[Bmim][Cl](fd) and Kevlar/PAM/[Bmim][Cl](fd)/PA membranes showed good tensile stress (0.8 and 0.6 MPa, respectively).

The composite membranes elaborated by spin-coating, LbL self-assembly, freeze drying, and vacuum-assisted flocculation techniques showed promising proton conductivity, thermal behavior, and mechanical stability, thus showing that these techniques are suitable for IL-based composite membrane preparation for PEMFC applications. However, further detailed studies are required.

## 5. Concluding Remarks

Today, the need to prepare IL-based composite polymer membranes for PEMFC applications in a wide range of temperatures is continuously increasing. For this purpose, different techniques are used to introduce the IL into polymer membranes with desirable physicochemical properties, namely, IL incorporation into a polymer solution, membrane swelling in the IL solution, and cross-linking. Despite the reported findings, the best method for IL introduction into polymer membranes is still under discussion because of the different limits of the mentioned methods. However, IL incorporation into a polymer solution is the most frequently used method due to the ease of process and rapid membrane formation. Nonetheless, the decrease in membrane mechanical stability and IL leaching present limitations of this method. Impregnated membranes with IL reveal good mechanical stability, but such membranes still suffer from IL leaching because of the lack of strong electrostatic interactions between IL and polymer chains. One of the possible ways to solve the problem of IL leaching is membrane cross-linking by covalent linkages between IL and polymer chains. However, the reduction in proton conductivity is the main limit of this method, owing to the decrease of IL ionic mobility. In addition, some recent techniques (such as layer-by-layer self-assembly, vacuum-assisted flocculation, spin coating, and freeze drying) can be also used for IL-based membrane preparation. Furthermore, the polymerization of ILs with polymerizable groups can be realized. The advantage of this last approach is the possibility of forming a continuous and uniform conducting network within the polymer membrane.

## Figures and Tables

**Table 1:** Summary of composite membranes produced by the incorporation of ILs into a polymer solution.

Membrane Composition	Solvent	Operating Temperature (°C)	Highest Proton Conductivity (S·cm^−1^)	Observations	Ref.
[BMI][HSO_4_]/SPEEK [Im][HSO_4_]/SPEEK [MI][HSO_4_]/SPEEK	NMP	25 and 80	150·10^−3^	It was found that by adding ILs, the surface roughness was reduced. The [MI][HSO_4_]/SPEEK composite membrane with 5 wt.% [MI][HSO_4_] showed the best proton conductivity.	[50]
Phosphonated IL-SBA-15/SPEEK	NMP	60 to 140	10.2·10^−3^	The composite membrane containing 6 wt.% of IL-SBA exhibited the highest tensile strength of 23 MPa. The maximum proton conductivity value was observed for the composite membrane with 6 wt.% of IL-SBA.	[30]
[TEA-PS][HSO_4_]/MOF/SPEEK [BImH][HSO_4_]/MOF/SPEEK [BMI][HSO_4_]/MOF/SPEEK	DMA	25 and 80	140·10^−3^	The [TEA-PS][HSO_4_]/MOF/SPEEK composite membrane with 2.5 wt.% of IL showed the highest conductivity at both operating temperatures (25 and 80 °C).	[51]
TIL_1_/SPSU TIL_2_/SPSU TIL_3_/SPSU	NMP	105 to 175	5.81·10^−2^	The membranes showed acceptable thermal stability. The highest conductivity value was measured for TIL_3_/SPSU with 1 mole ratio of TIL_3_ at 175 °C.	[52]
[BAIM][TFSI]/PI	Methylene chloride	25 to 160	1.0·10^−2^	The resultant membranes were thermally stable up to 350 °C. [BAIM][TFSI] demonstrated excellent conductivity of 5.6·10^−2^ S·cm^-1^ at 140 °C. It was found that the composite membrane containing 30 wt.% of IL showed the greatest tensile strength of 72.5 MPa.	[53]
[TEA-PS][HSO_4_]/PBI/SPEEK [BImH][HSO_4_]/PBI/SPEEK	DMA	25 and 80	101·10^−3^	The composite membrane containing 5 wt.% of [TEA-PS][HSO_4_] presented the best conductivity at 80 °C and 60% RH. The [TEA-PS][HSO_4_]/PBI/SPEEK composite membrane with 5 wt.% of IL exhibited the highest OCP and current density (0.97 V and 1.83 A·cm^−2^, respectively).	[54]
[dema][NTf_2_]/PBI [HHTMG][NTf_2_]/PBI [dema][NTf_2_]/PA/PBI [HHTMG][NTf_2_]/PA/PBI	DMAC	80 to 180	60·10^−3^	[HHTMG][NTf_2_]/PA/PBI presented the highest conductivity at 180 °C. The resultant composite membrane demonstrated great thermal stability up to 250 °C. However, the pristine PBI membrane showed better mechanical properties as compared to composite membranes.	[55]
NMPC/PVA PAN IL/NMPC/PVA SiO_2_/NMPC/PVA PAN IL/SiO_2_/NMPC/PVA	Water	25 to 100	1.54·10^−3^	The PAN IL/NMPC/PVA composite membrane with 20 wt.% PAN IL revealed the highest conductivity at 100 °C. The membranes were thermally stable up to 100 °C. The NMPC/PVA/SiO_2_ membrane showed better mechanical features as compared to PAN IL/NMPC/PVA membranes.	[56]
[C_1_Im][NTf_2_]/PBI [dema][TfO]/PBI [emim][TfO]/PBI [HOemim][NTf_2_]/PBI	DMF	100 to 250	108.9·10^−3^	The resultant IL/PBI composite membranes presented great thermal stability (between 310 and 383 °C). The maximum conductivity was observed for the [dema][TfO]/PBI composite membrane at 250 °C. The [dema][TfO]/PBI composite membrane demonstrated the best tensile strength of 7.8 MPa.	[35]
[BMIM][NCS]/PBI [BMIM][Cl]/PBI [BMIM][NTf_2_]/PBI [BMIM][I]/PBI [BMIM][PF_6_]/PBI	DMAC	0 to 200	9.4·10^−2^	The composite membranes showed good thermal stability at 200 °C. The [BMIM][BF_4_]/PBI composite membrane showed the highest proton conductivity at 200 °C. The [BMIM][Cl]/PBI membrane demonstrated the highest Young’s modulus (3.7 GPa) and tensile stress (141 MPa).	[57]
[EHNH_2_][H_2_PO_4_]/PVDF [Im][H*ex*]/PVDF	DMSO, DMC	20 and 60	0.15	The [EHNH_2_][H_2_PO_4_]/PVDF composite membrane showed the highest proton conductivity at 20 °C. By increasing the temperature, the proton conductivity of the imidazolium-based IL increased, whereas the proton conductivity of phosphated-based membranes decreased.	[58]
[dema][TfO]/PA/PVDF-HFP	Acetone	RT	6.3·10^−4^	The [dema][TfO]/PA/PVDF-HFP composite membrane with 40 wt.% of Il showed the maximum value for proton conductivity. Leakage of IL at high concentrations resulted in proton conductivity reduction.	[59]
[TEA-PS][HSO_4_]/Nafion^®^ [TEA-PS][BF_4_]/Nafion^®^ [TEA-PS][CF_3_SO_3_]/Nafion^®^	DMF	25 and 80	1.59	[TEA-PS][HSO_4_]/Nafion^®^ containing 5 wt.% of IL demonstrated the highest proton conductivity at 25 and 80 °C. The rise in operating temperature from 25 to 80 °C led to an increase in the IL leaching for all composite membranes.	[60]

**Table 2:** Summary of composite membranes prepared by the polymer impregnation with IL.

Membrane Composition	Operating Temperature (°C)	Highest Proton Conductivity (S·cm^−1^)	Observations	Ref.
[MIM][TFSI]/Matrimid^®^ [EIM][TFSI]/Matrimid^®^ [PIM][TFSI]/Matrimid^®^ [BIM][TFSI]/Matrimid^®^	25 to 150	1·10^−3^	The composite films containing [MIM][TFSI] showed the maximum proton conductivity value at 150 °C. The composite films exhibited the greatest thermal stability in the temperature range from 260 to 290 °C. The mechanical features of the composite membranes were better than those of pure Matrimid^®^.	[61]
[C_4_im][BEHP]/PI [C_4_im][DBP]/PI [C_1_im][DBP]/PI	25 to 115	2.0·10^−2^	The resultant composite membranes demonstrated great retention ability against IL leaching. It was found that [C_4_im][DBP])/PI composite film presented the highest proton conductivity at 115 °C.	[62]
[MeIm][Tf]/Polyoxadiazole/PVP	40 to 120	1.3·10^−3^	The [MeIm][Tf]/polyoxadiazole/PVP composite membranes showed a good degree of IL impregnation of 297%. The membrane samples demonstrated great thermal stability up to 350 °C. The composite films showed acceptable mechanical features.	[63]
[Vim][Tf]/PI [AIm][Tf]/PI [MIm][Tf]/PI [PVim][Tf]/PI [Vim][Tf]/[PVim][Tf]/PI	30 to 150	1.0·10^−4^	The [Vim][Tf]/[PVim][Tf]/PI composite membrane showed the highest Young’s modulus and elongation at break of 1371 MPa and 271%, respectively. The highest impregnation content was observed for the [Vim][Tf]/[PVim][Tf]/PI membrane (276 ± 16 wt.%). The composite films exhibited tunable thermal stability up to 300 °C.	[64]
[EMIM][ESO_4_]/ZrP/PTFE	200	0.061	The composite membrane showed great proton conductivity at 200 °C and non-humid conditions. The composite membrane was thermally stable as only 20% of weight loss was observed at 500 °C.	[65]
Dual acidic IL/ETFE	30 to 95	259·10^−3^	The membrane sample with higher IEC demonstrated better proton conductivity at the same operating condition. The composite membrane with the IEC of 3.4 meq·g^−1^ showed the highest conductivity at 95 °C. The resultant membranes were thermally stable up to 280 °C.	[66]
[HMIM][TCM]/ZrSi/GLY/PTFE [BMIM][SCN]/ZrSi/GLY/PTFE	25 and 200	0.196	The composite membrane showed higher proton conductivity than that of Nafion^®^ at 25 °C. It was found that the membrane proton conductivity increased by water uptake increasing. The composite membranes exhibited great thermal stability up to 200 °C.	[67]
ZrP/PTFE Lignin/ZrP/PTFE [HMIM][C_4_N_3_]/Lignin/ZrP/PTFE [DMEA][OMS]/Lignin/ZrP/PTFE [EMIM][CH_3_O_3_S]/Lignin/ZrP/PTFE	25 to 150	1.0·10^−1^	The hexyl-based, IL-based membrane showed the highest proton conductivity at 25 °C. The resultant composite membranes showed a good potential to be used in low-temperature PEMFC.	[68]

**Table 3:** Summary of composite films prepared using cross-linking.

Membrane Composition	Chemical Agents	Operating Temperature (°C)	Highest Proton Conductivity (S·cm^−1^)	Observations	Ref.
P[VBIm][Cl]/NbPBI P[MPIm][Br]/NbPBI P[TPAm][Br]/NbPBI	Solvent and initiator: DMAC, AIBN	110 to 170	0.074	FTIR analysis confirmed that PILs are chemically cross-linked to polymer. The NbPBI/P[MPIm]Br composite film demonstrated the highest proton conductivity at 170 °C. The composite film showed good thermal stability (∼220 °C).	[70]
P[TSPDO][BrCl]/NbPBI	Solvent and initiator: DMSO, AIBN	110 to 170	0.061	Modified membranes demonstrated higher thermal stability (∼300 °C) as compared to pure membranes (∼250 °C). The composite polymer film with 30 wt.% of P[TSPDO][BrCl] showed the greatest conductivity at 170 °C. The cross-linked composite membranes exhibited good mechanical properties.	[71]
[APMIm][Br]-GO/[MIm][TfO]/PSAN	Solvent and photo-initiator: [MIm][TfO], benzoin isobutyl ether	100 to 160	1.48·10^−2^	The membrane containing 1 wt.% of [APMIm][Br]-GO demonstrated the best proton conductivity at 160 °C. It was found that the addition of [APMIm][Br]-GO caused a decrease in the membrane mechanical properties. The leaching test showed that by increasing the concentration of [APMIm][Br]-GO, leaching of PrIL was reduced.	[72]
PIL(PBI-BF_4_)/PBI PIL(PBI-BF_4_)/PA/PBI	Solvent and cross-linker: DMAC, γ-(2, 3-epoxypropoxy) propyltrimethoxysilane	110 to 170	0.117	To increase the conductivity of membranes, some samples were immersed in a PA solution. The membrane containing 40 wt.% of PIL exhibited the maximum value of proton conductivity at 170 °C. An increase in concentration of PIL caused a decrease in the mechanical features of composite films.	[73]
[HSO_3_-BVIm][TfO]/MMA [HSO_3_-BVIm][TfO]/hPFSVE	Cross-linker and photo-initiator: glycerol dimethacrylate, 2-hydroxy-2-methyl propiophenone	25 to 90	1.0·10^−2^	FTIR confirmed the photochemical copolymerization reaction between IL and both hPFSVE and MMA. Membranes revealed acceptable proton conductivity in both dry and wet states. The membranes represented acceptable thermal stability (≥200 °C).	[74]
[CPDOc]Br_2_/PBI [CPDOc]Br_2_/PA/PBI	Solvent and initiator: DMSO, AIBN	110 to 170	0.121	The [CPDOc]Br_2_/PA/PBI composite membrane with 30 wt.% of IL showed the best conductivity at 170 °C. The resultant films were thermally stable up to 200–250 °C.	[75]

## Data Availability

No new data were created or analyzed in this study. Data sharing is not applicable to this article.

## Abbreviations

AFC	Alkaline fuel cell
AIBN	Azobis-(isobutyronitrile)
DMA	*N,N*-dimethylacetamide
DMAC	Dimethylacetamide
DMF	*N,N*-dimethylformamide
DMFC	Direct methanol fuel cell
DMSO	Dimethyl sulfoxide
ETFE	Ethylene-*co*-tetrafluoroethylene
fd	Freeze drying
FFC	Field flow channel
FIL	Functionalized ionic liquid
FTIR	Fourier-transform infrared spectroscopy
GDL	Gas diffusion layer
hPFSVE	Hydrolyzed perfluoro-3,6-dioxa-4-methyl-7-octene sulfonyl fluoride
IEC	Ion exchange capacity
IL	Ionic liquid
LbL	Layer by layer
MCFC	Molten carbonate fuel cell
Meq	Milliequivalent
MMA	Methyl methacrylate
MOF	Metal organic framework
Nb	Norbornene
NMP	*N*-methyl-2-pyrrolidinone
NMPC	*N*-methylene phosphonic chitosan
OCNT	Carbon nanotube oxide
PA	Phosphoric acid
PAFC	Phosphoric acid fuel cell
PAN	Propyl ammonium nitrate
PBI	Polybenzimidazole
PEM	Polymer electrolyte membrane
PEMFC	Proton exchange membrane fuel cell
PI	Polyimide
PIL	Polymerized ionic liquid
PrIL	Protic ionic liquid
PSAN	Poly(styrene/acrylonitrile)
PSP	Perfluoro-sulfonated polymers
PTFE	Polytetrafluoroethylene
PU	Polyurethane
PVA	Poly(vinyl alcohol)
PVP	Polyvinylpyrrolidone
RH	Relative humidity
SBA-15	Santa Barbara Amorphous-15
SBES	Polystyrene-*block*-poly(ethylene-*ran*-butylene)-*block*-polystyrene
SEM	Scanning electron microscope
SILM	Supported ionic liquid membrane
SiO_2_	Silicon dioxide
SOFC	Solid oxide fuel cell
SPEEK	Sulfonated poly ether ether ketone
SPSU	Sulfonated polysulfone
TGA	Thermogravimetric analysis
TIL	Triazole-based IL
ZrP	Zirconium phosphate
